# Integrating remote sensing and multi-criteria decision analysis for groundwater zoning in the Eastern Desert of Egypt

**DOI:** 10.1038/s41598-026-55824-y

**Published:** 2026-06-12

**Authors:** H. Ezz

**Affiliations:** 1 Civil Engineering Department, National Research Centre, Engineering & Renewable Energy Research Institute, Giza, Egypt; 2 Al Madinah High Institute for Engineering and Technology, Giza, Egypt

**Keywords:** Sustainable groundwater management, Groundwater potential zones, Remote sensing, GIS, AHP method, Multi-criteria decision analysis (MCDA), Egypt, Environmental sciences, Hydrology, Solid Earth sciences

## Abstract

The Eastern Desert of Egypt is a hyper-arid region where groundwater serves as the primary alternative to surface water for sustainable development. However, comprehensive assessments of groundwater potential across this vast and geologically complex region remain limited. This study addresses this gap by developing a spatial model for delineating groundwater potential zones (GWPZ) using an integrated approach that combines remote sensing, Geographic Information Systems (GIS), and the Analytical Hierarchy Process (AHP). Seven key thematic layers, precipitation, lithology, slope, drainage density, soil type, land use/land cover (LULC), and lineament density, are selected, standardized, and weighted based on hydrogeological relevance. These layers are integrated through a weighted overlay analysis in a GIS environment. The resulting GWPZ map is initially classified into five categories: very high, high, moderate, low, and very low potential. The very high and very low potential zones have very small areas in the final output due to the region’s arid conditions and hydrogeological limitations. The remaining zones covered the study area as follows: high potential (7.7%), moderate (54.5%), and low potential (37.7%). Model reliability is assessed through two complementary validation approaches. First, 370 groundwater wells with available location data are spatially overlaid on the GWPZ map, showing limited overlap with high recharge zones, as most wells target deep fossil aquifers not influenced by present-day surface conditions. Second, a supplementary validation using three independent surface-derived indicators: Topographic Wetness Index, curvature, and lineament–stream intersection density, demonstrated strong agreement with the GWPZ output. The integration of these two validation methods confirms the robustness of the model for mapping shallow groundwater recharge potential in arid environments. This framework offers a scalable, data-driven approach to support groundwater exploration and strategic water resource planning in similar regions worldwide.

## Introduction

Egypt, situated within the hyper-arid belt of North Africa, faces one of the most pressing water scarcity challenges in the world. With over 95% of the country receiving less than 100 mm of rainfall annually, the nation relies heavily on the Nile River as its primary freshwater source in addition to groundwater as a second source of water^1^. However, the sustainability of the Nile is increasingly under threat due to rapid population growth, rising agricultural demands, industrial development, and transboundary pressures. Groundwater, therefore, represents a critical supplementary resource, particularly in regions distant from the Nile Valley, such as the Eastern Desert. According to the Food and Agriculture Organization (FAO), Egypt’s annual renewable water resources per capita have fallen well below the absolute scarcity threshold of 500 cubic meters, classifying it as a country experiencing absolute water scarcity^2,3^. Compounding this crisis are the growing uncertainties posed by climate change, including elevated evapotranspiration, erratic rainfall, and declining upstream inflows, which collectively call for alternative and sustainable water sources.

In hyper-arid regions such as Egypt’s Eastern Desert, where rainfall is scarce and evapotranspiration rates are high, securing adequate water supplies is a persistent challenge. As surface water sources are extremely limited, groundwater becomes the primary and most viable source for meeting the needs of human consumption, agriculture, and development. Therefore, its identification and sustainable management are essential for regional water security. Accurate and up-to-date mapping of groundwater potential zones (GWPZ) is crucial for supporting informed decision-making in water resource management and for guiding future development activities in this region^4^. Traditional methods of groundwater exploration, often relying on costly and time-consuming ground-based surveys, can be limited in their spatial coverage and efficiency^4,5^.

In recent decades, the integration of Geographic Information Systems (GIS) and remote sensing technologies has emerged as a powerful and cost-effective approach for assessing and mapping natural resources, including groundwater potential^5–9^. Remote sensing provides valuable spatial data on various environmental factors that influence groundwater occurrence, such as precipitation intensities, lithology, surface slopes, lineaments, drainage patterns, vegetation cover, and land surface characteristics. To effectively integrate these diverse factors in assessing groundwater potential, multi-criteria decision analysis (MCDA) methods, such as the AHP, have proven to be highly suitable^10–15^. The AHP method, developed by^16^, provides a structured framework for evaluating and weighting various criteria based on their relative importance in influencing a particular phenomenon, such as groundwater potential. Previous studies in Egypt have focused on mapping GWPZ in specific areas of the Eastern Desert, such as the Wadi Qena Basin^5,17–19^, the Wadi Hodein area^20^, and the central Eastern Desert^9,21–24^.

Despite these advancements, there remains a significant knowledge gap regarding the groundwater potential of the Eastern Desert. However, most of the studies have focused on relatively smaller watersheds or specific regions within the Eastern Desert, with few efforts aimed at large-scale, multi-factorial groundwater assessments in the basement-dominated desert regions. No study has comprehensively mapped the entire Eastern Desert of Egypt, despite its vast size and critical importance for future water resource management and sustainable development. Given the region’s tectonic complexity, heterogeneous lithology, and episodic hydrological inputs, a tailored GIS-AHP framework is essential for developing an effective and practical groundwater potential map.

In this context, the selection of appropriate thematic layers is paramount. Based on hydrological theory, regional experience, and previous modeling efforts, this study considers seven critical factors: precipitation, lithology, slope, land use/land cover (LULC), drainage density, soil type, and lineament density. Each of these parameters is known to significantly influence groundwater recharge, storage, or movement. For instance, rainfall provides the primary input for recharge, while geology and lineament density govern subsurface permeability and storage potential. Slope and drainage density regulate runoff and infiltration, whereas soil and land cover modulate infiltration rates and surface retention.

The main objective of this study is to identify and map GWPZ in the Eastern Desert of Egypt using an integrated GIS-AHP model. The approach involves reclassifying and standardizing seven thematic datasets, followed by applying AHP to derive factor weights, which are then combined using weighted overlay analysis. The resulting map aims to support sustainable groundwater exploration, water resource planning, and policy development, especially in rural and infrastructure-scarce regions of Egypt. In doing so, the study contributes to Egypt’s broader water security goals and provides a methodological framework applicable to other arid regions facing similar challenges.

## Study area

**Fig. 1:**
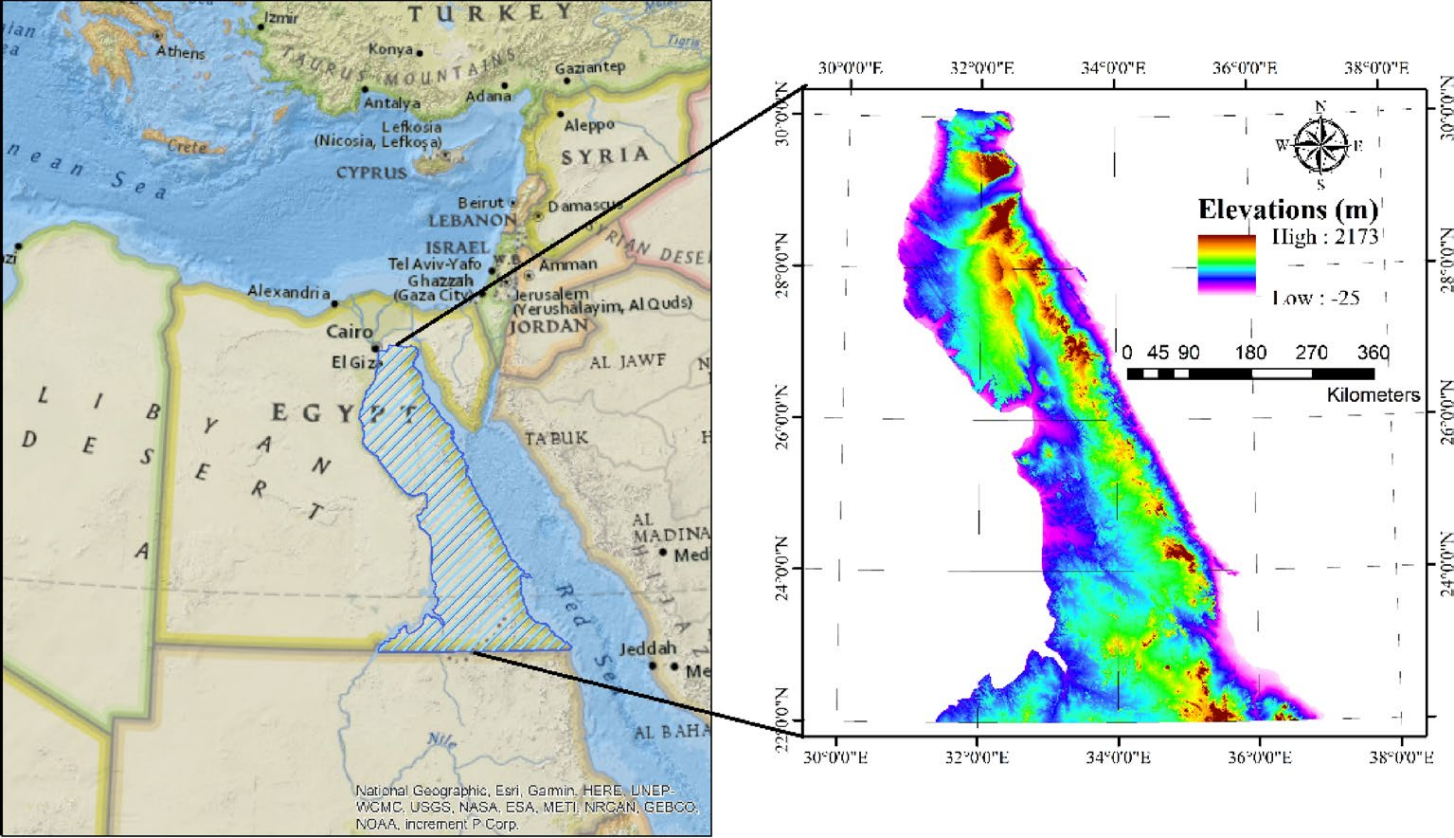
Study area of the Eastern Desert of Egypt.

The Eastern Desert of Egypt extends from the Nile Valley in the west to the Red Sea in the east, and from the Sudanese border in the south to the northern fringes of the Nile Delta (Fig. 1). It encompasses a diverse and rugged landscape dominated by arid plains, high-relief mountains, and numerous wadis (dry riverbeds) that act as ephemeral drainage pathways. The region is part of a hyper-arid climatic zone, characterized by extremely low and erratic rainfall (less than 30 mm annually), intense evaporation rates, and scarce surface water bodies^2,25^. Under these conditions, groundwater remains the primary and often sole source of freshwater for domestic, agricultural, and developmental uses.

The topography features the Red Sea Hills, an uplifted mountain chain running parallel to the Red Sea coast, with elevations exceeding 2,000 m. The terrain slopes westward toward the Nile Valley, forming a series of dissected plateaus and undulating plains. This geomorphology plays a crucial role in controlling runoff, infiltration, and groundwater recharge dynamics. Socio-economically, the region hosts scattered human settlements, mining operations, tourism infrastructure, and limited agriculture sustained mainly by accessible groundwater sources. As demand rises due to population growth and regional development initiatives, the strategic management of groundwater resources becomes increasingly important^1^.

Hydrogeologically, the Eastern Desert is underlain by three major aquifer systems: the Nubian Sandstone Aquifer in the west, fractured basement aquifers in the central and southern zones, and coastal aquifers along the Red Sea margin. Recharge to these aquifers is highly constrained and primarily occurs through episodic flash floods and localized wadi infiltration events^1,9^. Structural features such as fractures and fault zones greatly enhance the potential for groundwater storage in otherwise low-porosity formations.

The Eastern Desert of Egypt comprises a tectonically complex terrain dominated by three major geological units:

- The Precambrian crystalline basement complex, which occupies much of the central and southern regions, is composed of granitic intrusions, metavolcanics, and schists. These units are characterized by low primary porosity but often exhibit enhanced permeability through fractures and shear zones^26,27^.
- The Phanerozoic sedimentary sequences, mainly Cretaceous–Tertiary sandstones and limestones, are prevalent in the north and southeastern parts. These formations often act as productive aquifers, especially when structurally disturbed^28^.
- Quaternary alluvial deposits and wadi fill sediments, particularly near the Red Sea foothills and Gebel Elba, are typically unconsolidated and serve as potential shallow aquifers^19,20^.

Soil types across the region also exhibit spatial variability. Arenosols and Regosols, common in sandy and rocky zones, offer high infiltration capacity, making them favorable for groundwater recharge^29^. Fluvisols, often found near wadi systems and coastal plains, support recharge due to their alluvial origin. Conversely, Calcisols and Ferralsols tend to restrict infiltration due to compaction and low permeability^30^.

These geological and pedological characteristics significantly influence recharge, storage, and the spatial distribution of groundwater potential in the Eastern Desert^9,21^.

## Material and methods

This study employs an integrated geospatial methodology that combines remote sensing, multi-source geodata, and the AHP within a GIS environment to delineate GWPZ in the Eastern Desert of Egypt. This approach enables the systematic evaluation of multiple hydrogeological and environmental parameters, each of which contributes to the spatial variability of groundwater occurrence and recharge.

The methodology is structured into three major phases: (1) data acquisition and preparation, (2) thematic map generation and classification, and (3) multi-criteria integration using AHP-based weighted overlay analysis. A total of seven key thematic layers, precipitation, geology (lithology), slope, drainage density, soil type, LULC, and lineament density are selected based on their relevance to groundwater dynamics in arid environments.

Geospatial datasets are first standardized through georeferencing and projection into a common spatial framework (UTM). Derived products such as slope and lineament density are extracted from high-resolution Digital Elevation Models (DEMs), while other layers are prepared using satellite imagery, geological surveys, and global datasets. Each thematic map is then reclassified into five groundwater potential categories ranging from 1 (very high potential) to 5 (very low potential), based on their hydrogeological characteristics.

**Fig. 2:**
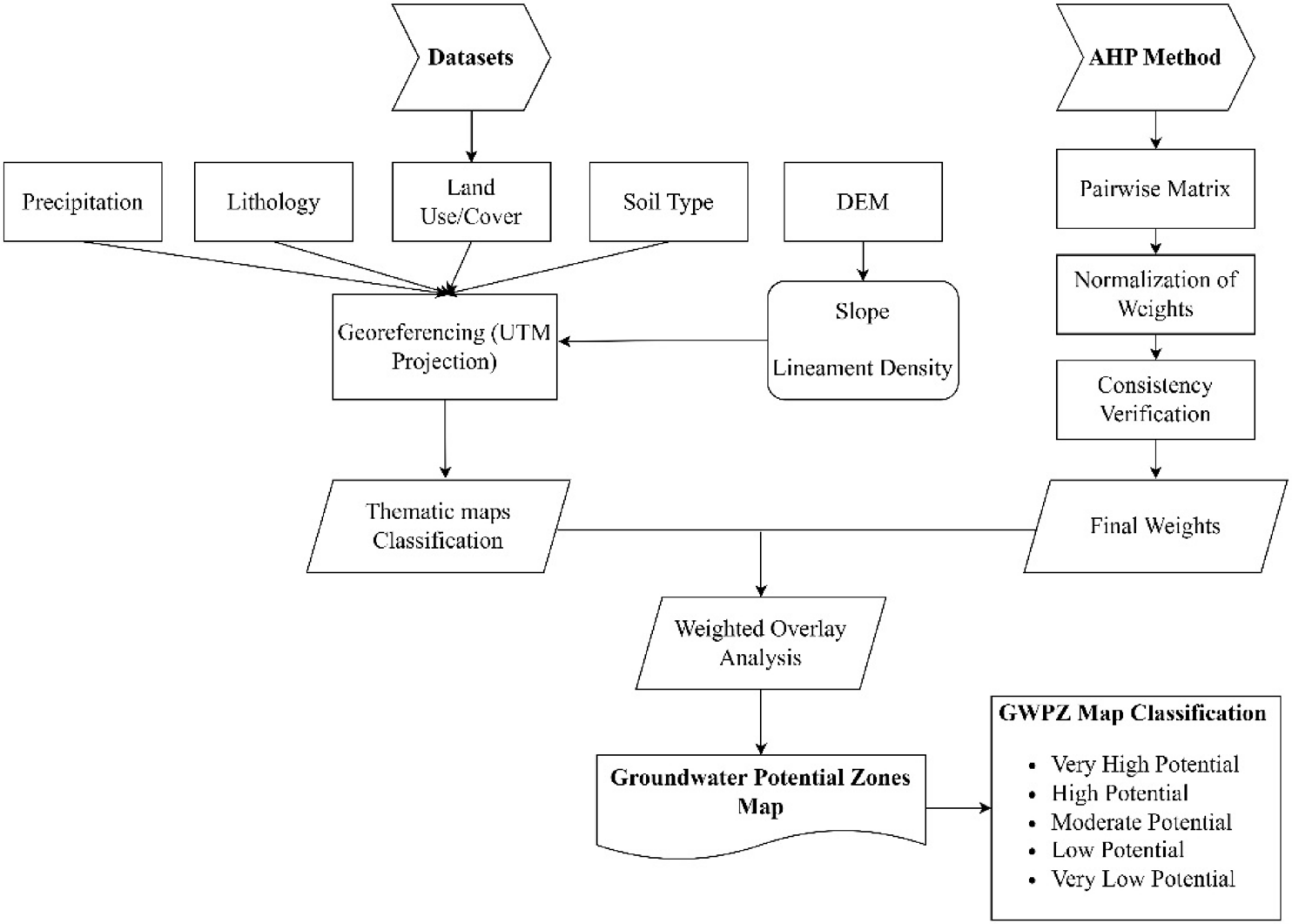
Flow chart of the methodology used for GWPZ Mapping.

In parallel, the AHP method is applied to derive the relative importance (weights) of each factor through pairwise comparison, normalization, and consistency verification. These weights are then applied in the weighted overlay analysis, which resulted in a composite GWPZ index map. The final map is further classified into five classes, from very high to very low groundwater potential zones. The overall methodology is summarized in Fig. 2, which illustrates the flow from dataset processing to GWPZ map generation. To validate the resulting GWPZ map, two complementary approaches are applied. First, a spatial overlay analysis is conducted using 370 existing groundwater well locations distributed across the Eastern Desert, evaluating the correspondence between well positions and predicted groundwater potential classes. Second, a supplementary validation is performed using three independent surface-derived indicators, Topographic Wetness Index (TWI), curvature, and lineament–stream intersection density, each classified into groundwater favorability classes consistent with the GWPZ map. These datasets are compared with the GWPZ output using a Python-based validation script that computed multiple spatial agreement and statistical association metrics. Together, these two validation approaches provided a comprehensive assessment of the model’s reliability and predictive performance.

### Parameters for groundwater potential mapping

The selection of appropriate thematic parameters is a fundamental step in developing accurate GWPZ maps, particularly in arid and structurally complex regions such as the Eastern Desert of Egypt. This study incorporates seven geospatial layers: precipitation, lithology, slope, drainage density, LULC, soil type, and lineament density. These parameters are selected based on their hydrogeological significance, spatial availability, and proven application in previous studies in the Eastern Desert.

The influence of proximity to the Nile Valley is excluded from this study, as numerous investigations have established that lateral groundwater seepage from the Nile into the eastern desert fringes is limited to a few kilometers and rarely exceeds 5 km^31–33^.

#### Precipitation

**Fig. 3:**
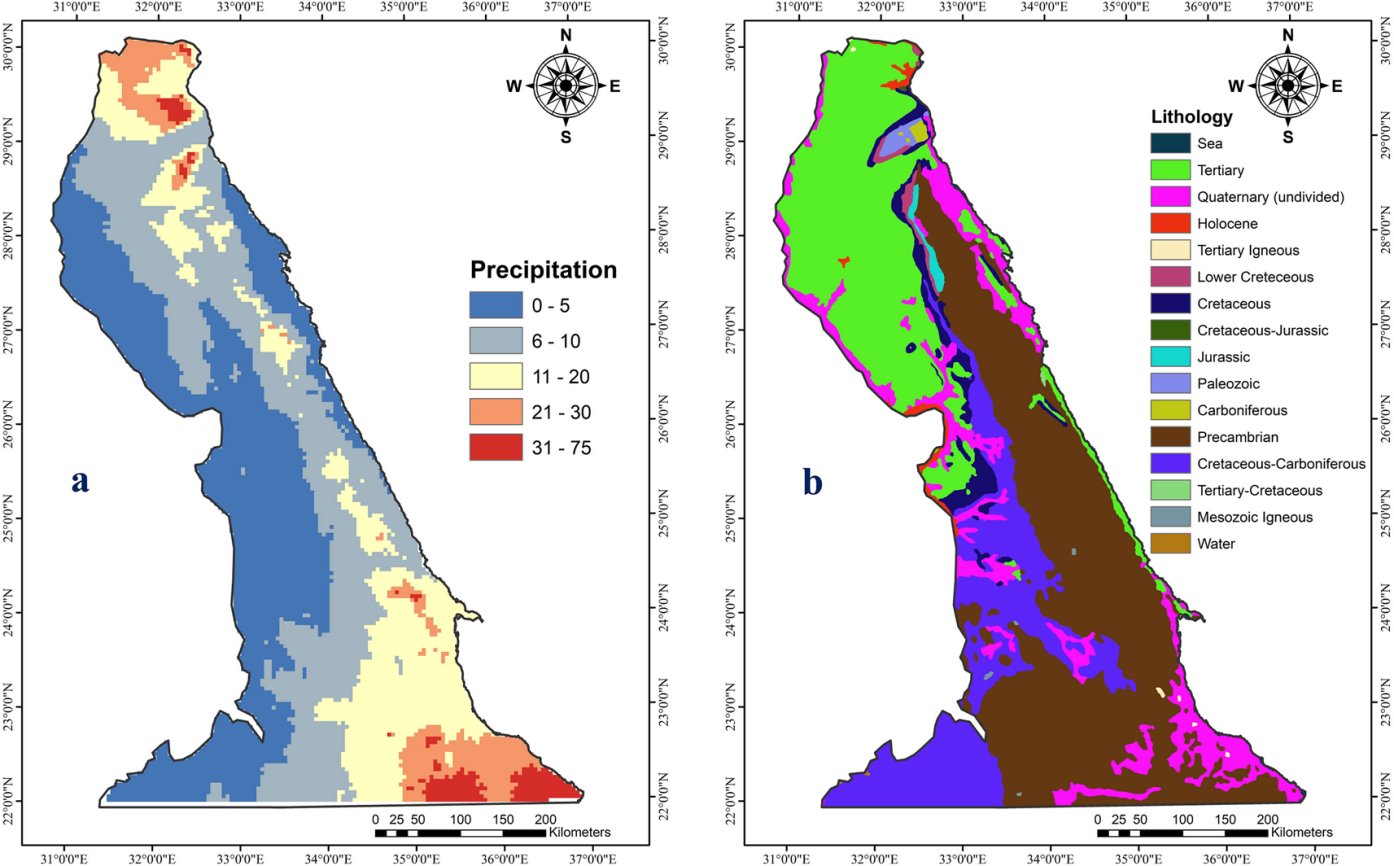
(**a**) Precipitation rates, and (**b**) Lithology of the study area.

Rainfall is a key driver of natural groundwater recharge; particularly where other sources are absent. In hyper-arid environments such as Egypt’s Eastern Desert, precipitation is both limited and erratic; however, occasional intense rainfall events, especially in mountainous areas, can lead to substantial infiltration through wadis, fractured bedrock, and ephemeral channels^34,35^. Regions that receive relatively higher rainfall are generally more favorable for groundwater accumulation. In this study, historical rainfall data spanning the period from 2010 to 2020 is collected to assess spatial variability across the study area as illustrated in Fig. 3a. The dataset is derived from the CRU-TS-4.06 gridded product by the Climatic Research Unit, University of East Anglia, and downscaled using WorldClim 2.1 to improve spatial resolution and apply bias correction^36^. The native resolution of the precipitation grids is 2.5 arc-minutes (~ 4.5 km ≈ 21 km^2^ per pixel); these rasters are bilinearly resampled to the study’s common 30 m working grid to match the other thematic layers. Annual averages are computed to represent mean rainfall depths across the region.

#### Lithology

Geological formations play a fundamental role in controlling the subsurface storage and movement of groundwater. Lithologies such as fractured basement rocks and porous sedimentary units, particularly sandstone and limestone, tend to possess higher permeability and porosity, making them more conducive to groundwater accumulation^9,21,37^. In contrast, compacted or crystalline formations, including shale and igneous rocks, typically act as barriers to groundwater flow due to their low hydraulic conductivity. In this study, geological formations, Fig. 3b, are delineated and classified using geological maps provided by United States Geological Survey^38^ based on their anticipated influence on groundwater occurrence and transmissivity. The source map has a native cartographic scale of 1:250 000 (minimum mapping unit ≈ 250 m); vector polygons are rasterized and resampled to the common 30 m working grid to align with the other thematic layers.

#### Topographic data (Slope)

**Fig. 4:**
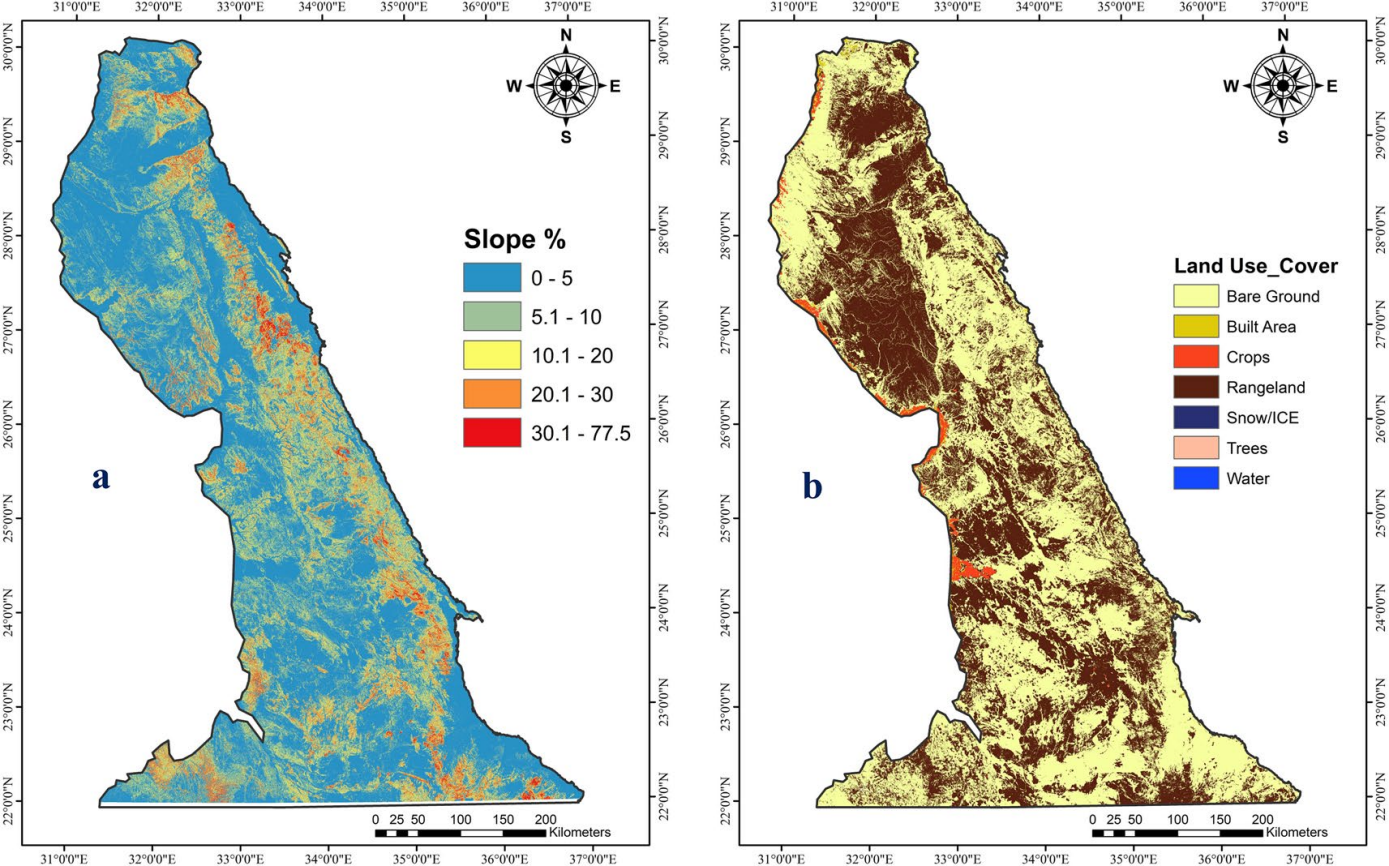
(**a**) Slope, and (**b**) LULC of the study area.

Slope controls the partitioning between infiltration and surface runoff. Gentle slopes favor water retention and percolation, thereby enhancing recharge, whereas steep slopes promote rapid runoff and erosion, reducing the potential for infiltration. Slope gradients are derived from a Digital Elevation Model (DEM) using standard terrain analysis techniques and classified according to recharge potential. The Digital Elevation Model (DEM) for Egypt’s Western Desert has a resolution of 1 arc-second (about 30 m) and is geo-referenced to UTM WGS84 Zone 36 North. Figure 4a shows how this Digital Elevation Model (DEM) is made using ArcGIS and the Spatial Analyst Extension to get data from the ALOS World 3D—30m (AW3D30) version 2.1^39^.

#### Drainage density

Drainage density is a widely used hydrological parameter that serves as an indirect indicator of surface permeability and infiltration capacity. It is defined as the total length of drainage channels per unit area and provides insight into the terrain’s ability to absorb or shed surface water. Generally, areas with low drainage density indicate higher infiltration potential due to more permeable surface materials, while high drainage density often corresponds to compacted or impermeable substrates that promote surface runoff and limit groundwater recharge^21,40,41^.

**Fig. 5:**
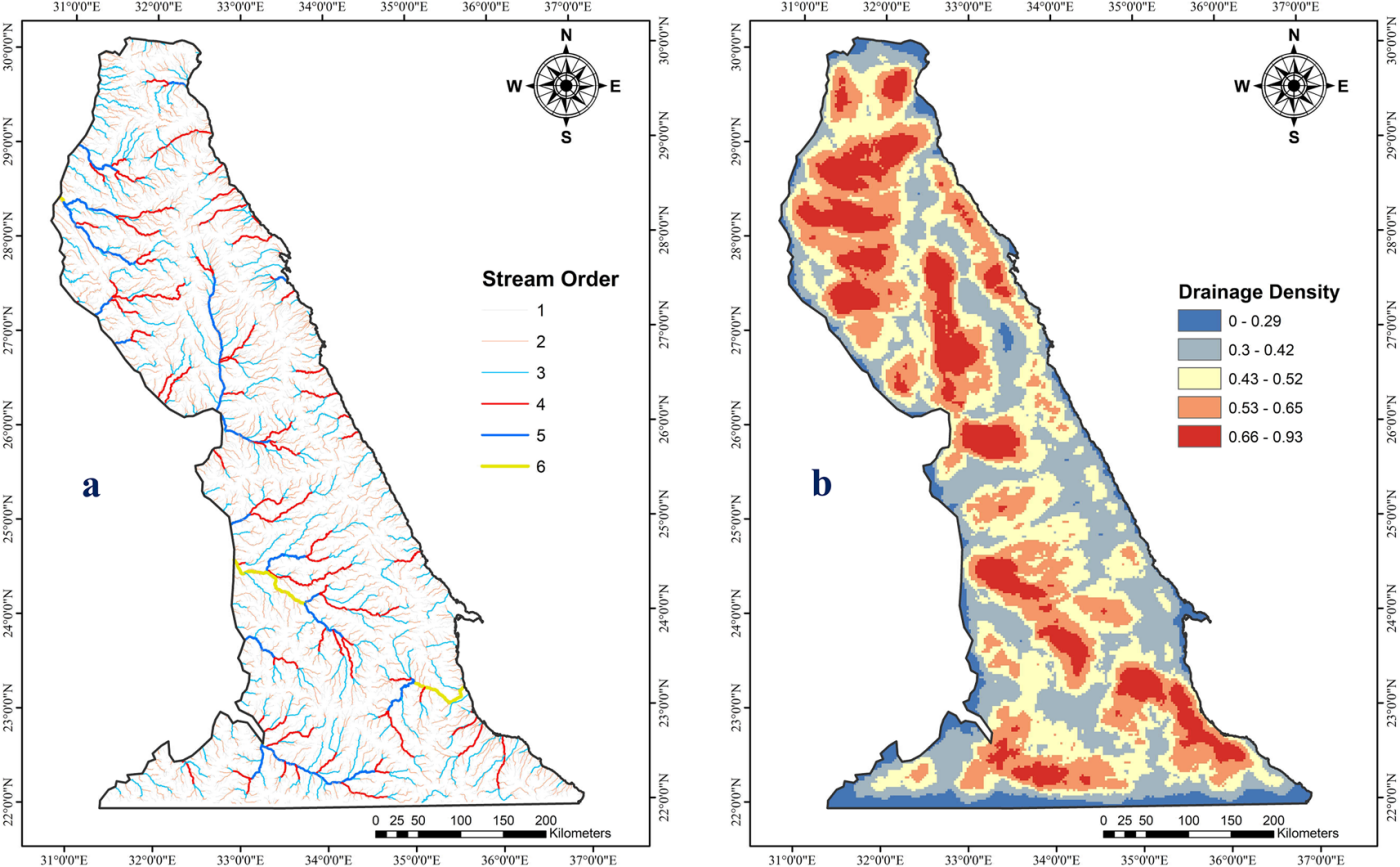
(**a**) Stream network and orders, and (**b**) Drainage density of the study area.

In this study, drainage density is derived through hydrological modeling in a GIS environment using a high-resolution Digital Elevation Model (DEM). The DEM is first preprocessed by applying flow direction and flow accumulation algorithms to delineate the drainage network. Stream segments are classified according to the Strahler stream order system to distinguish between primary and higher-order channels (Fig. 5a). This hierarchical classification allowed for the identification of major flow paths and their distribution across the landscape. Once the drainage network is fully extracted and validated, the total stream length within each spatial unit is computed, and drainage density is calculated using a raster-based method. The resulting drainage density map offers a spatially continuous representation of infiltration potential across the study area, with distinct variations correlating to terrain features and geological controls. Figure 5b displays the drainage network overlaid with stream orders and the derived drainage density classes, highlighting zones with differing hydrological responses and recharge capacities.

#### Land use /Land cover (LULC)

LULC plays a critical role in shaping surface hydrology and influencing infiltration rates. Vegetated areas, such as agricultural fields and shrublands, enhance infiltration by reducing surface runoff and promoting soil moisture retention, thereby supporting groundwater recharge. In contrast, impervious surfaces like urban developments and exposed barren lands restrict percolation, leading to increased runoff and reduced recharge potential. In this study, LULC data are sourced from the Environmental Systems Research Institute (ESRI) global land cover map, a widely recognized and reliable dataset in GIS-based environmental assessments (Fig. 4b). ESRI is a global leader in geospatial software and provides high-quality spatial data that are essential for accurate hydrological and land surface analysis^42^. The ESRI global land-cover raster has a native spatial resolution of 10 m; it is resampled to the common 30 m working grid to ensure consistency with the other thematic layers used in this study.

#### Soil type

**Fig. 6:**
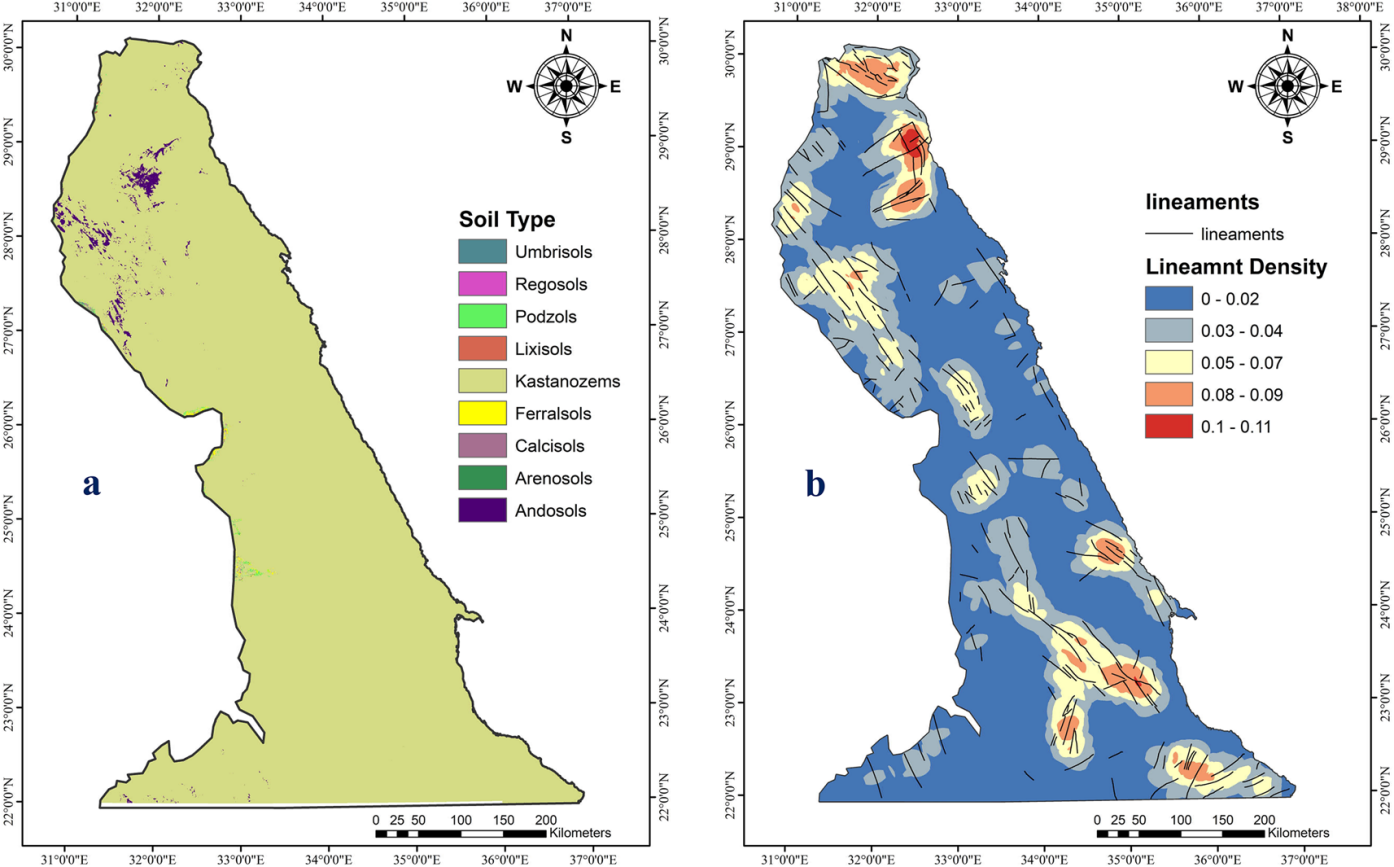
(**a**) Soil type, and (**b**) Lineament density of the study area.

Soil characteristics, particularly texture and structure, play a vital role in determining infiltration capacity and soil water retention (Fig. 6a). Well-drained soils such as sandy and loamy types typically exhibit high permeability, allowing for greater vertical percolation and enhancing groundwater recharge. In contrast, clay-rich soils have lower permeability and tend to retain surface water, limiting infiltration into deeper layers. In this study, soil data are obtained from globally validated sources, including the works of Hengl et al.^29^ and Shangguan et al.^30^, which provide high-resolution, harmonized soil property datasets suitable for spatial hydrological modeling. The dataset is supplied at a native 250 m grid spacing and is bilinearly resampled to the study’s common 30 m working grid to maintain consistency with the other thematic layers.

#### Lineament density

Lineaments, such as faults, fractures, and shear zones, play a critical role in facilitating groundwater movement, particularly in crystalline and hard rock environments where primary porosity is minimal^43^. In structurally complex regions like Egypt’s Eastern Desert, these features significantly enhance secondary porosity and serve as preferential pathways for infiltration and subsurface flow. Lineament patterns are extracted through remote sensing techniques and geological interpretation of satellite imagery, then processed in ArcGIS to generate a lineament density map. This map quantifies the concentration of structural features per unit area, offering a valuable proxy for fracture connectivity. The lineament inventory is digitized from Landsat-8 OLI false-colour composites and a 30 m shaded-relief DEM; the resulting density raster is produced on a 30 m grid and is therefore already congruent with the study’s common 30 m working resolution. Figure 6b illustrates the spatial distribution of lineament density across the study area.

Table 1 summarizes the source, original resolution or map scale, and the standardized raster resolution used for each of the seven thematic layers applied in the GWPZ model.

**Table 1:** Metadata summary of thematic input layers.

Thematic layer	Data source	Original Resolution / scale	Standardized resolution	Reference year
Precipitation	CRU-TS v4.06 + WorldClim 2.1 downscaling (2010–2020)	2.5 arc-min (~ 4.5 km)	30 m	2010 -2020
Lithology	USGS Geological Maps (1:250,000)	1:250,000 scale (vector)	30 m (rasterized)	2002
Slope	ALOS World 3D – AW3D30 v2.1	30 m	30 m	2021
Drainage Density	Derived from ALOS DEM using hydrological modeling	30 m	30 m	2021
LULC	ESRI Global Land Cover (2020)	10 m	30 m (resampled)	2020
Soil Type	SoilGrids v2	250 m	30 m (resampled)	2017
Lineament Density	Digitized from Landsat-8 OLI & shaded-relief DEM (manual interpretation)	~ 1:100,000 mapping scale	30 m	2022

### Analytical hierarchy process (AHP) for groundwater potential mapping

AHP is a structured decision-making technique introduced by^16^ that enables the integration of multiple spatial and thematic parameters by assigning relative weights based on expert judgment and pairwise comparison. AHP is a commonly adopted approach for multi-criteria site suitability assessments in numerous fields^44–48^. In the context of groundwater potential mapping, AHP has become a widely accepted method for synthesizing hydrogeological, topographical, climatic, and environmental data into a coherent spatial model.

In this study, AHP is applied to evaluate and prioritize seven parameters influencing groundwater occurrence in the Eastern Desert of Egypt. These parameters include precipitation, lithology, slope, drainage density, LULC, soil type, and lineament density. The weights assigned to each parameter are derived from a normalized pairwise comparison matrix in which each criterion is compared with the others using a 1–9 scale (1 = equal importance, 9 = extreme importance). These comparisons are used to calculate weights for each criterion, representing their relative influence on the goal. The process ensures consistency through the Consistency Ratio (CR), which should be less than 0.1 for reliable results. The pairwise comparisons and resulting weights were established based on published literature and the hydrogeological characteristics of the Eastern Desert of Egypt.

### Mapping controlling factors

To effectively implement the AHP using a weighted overlay in ArcGIS, the dataset is classified each into five categories (very high potential, high potential, moderate potential, low potential, and very low potential). This classification ensures a structured evaluation of multiple factors that influence spatial analysis, such as precipitation, lithology, slope, drainage density, LULC, soil type, and lineament density. By standardizing these parameters, it is possible to systematically assign weights and rank their influence on the study area, facilitating a more data-driven decision-making process.

Each dataset is classified based on its specific characteristics. For instance, precipitation values are categorized according to rainfall intensity, while slope classification followed terrain steepness. Similarly, geological formations, land cover types, soil classes, and lineament density are grouped based on their impact on hydrological and environmental processes. The following context will discuss the classification of each dataset in detail.

#### Precipitation classification

Precipitation data, illustrated in Fig. 3a, is categorized into five classes based on daily rainfall intensity (mm/day). The lowest category, 0–5 mm/day, represents arid or semi-arid regions with minimal rainfall. Areas receiving between 6–10 mm/day fall into the second category, indicating slightly wetter conditions. Moderate precipitation zones, ranging from 11–20 mm/day, form the third class. Higher rainfall intensities of 21–30 mm/day characterize the fourth category, while the highest precipitation class, more than 31 mm/day, corresponds to regions experiencing significant daily rainfall. This classification helps assess the potential influence of precipitation on surface runoff and erosion. This classification approach follows rainfall-based groundwater potential mapping methods adopted in arid regions^49^.

#### Lithology classification

The geological formations in the study area, as shown in Fig. 3b, are reclassified based on their hydrogeological properties, primarily porosity, permeability, and their capacity to facilitate groundwater recharge and storage. Lithological units such as Quaternary (undivided) and Holocene deposits are categorized as having very high groundwater potential, due to their unconsolidated nature and high infiltration capacity. Formations like the Tertiary, Tertiary-Cretaceous, and Carboniferous units are assigned high potential status, as they generally possess moderate porosity and may contain secondary permeability through fracturing or weathering. Sedimentary units including the Cretaceous, Lower Cretaceous, and Jurassic formations are considered to have moderate groundwater potential, as their groundwater occurrence is commonly controlled by bedding planes, weathering, and fracture-related secondary permeability, while their recharge and storage capacity remain lower than that of unconsolidated Quaternary deposits and highly permeable sandstone-dominated units^9,21,50^. More compact formations such as the Cretaceous-Jurassic and Cretaceous-Carboniferous sequences are classified as having low potential, due to their typically reduced permeability. Finally, very low groundwater potential is attributed to Precambrian basement rocks, Mesozoic Igneous, and Tertiary Igneous formations, which are generally impermeable and lack sufficient secondary porosity except where structurally disrupted. The hydrogeological behavior of lithological units in arid and semi-arid zones has been similarly classified in works^9,26^, who studied lithology-groundwater relationships in the Central Eastern Desert of Egypt. This classification scheme reflects both regional hydrogeological knowledge and established principles regarding lithologic influence on groundwater occurrence. It supports the broader AHP-based groundwater modeling by providing a geologically informed thematic layer for integration with other spatial parameters.

#### Slope classification

Slope is a key topographic factor influencing surface runoff and infiltration, both of which are critical in determining groundwater recharge potential. As shown in Fig. 4a, the slope of the study area is derived from a high-resolution Digital Elevation Model (DEM) and classified into five categories based on gradient. These categories are then reinterpreted in the context of groundwater favorability. Areas with gentle slopes (0–5%) are classified as having very high groundwater potential, as they favor water accumulation and infiltration with minimal runoff. Zones with moderate slopes (5.1–10%) are assigned high potential, reflecting their suitability for moderate infiltration while still maintaining limited runoff. Slopes ranging from 10.1% to 20% are considered to have moderate potential, as water retention begins to diminish and runoff increases. Steeper slopes between 20.1% and 30% are classified as low potential, due to rapid runoff and limited infiltration time. Finally, areas with very steep slopes (more than 30.1%) are assigned very low potential for groundwater recharge, as these terrains facilitate fast surface water discharge and are typically erosion-prone. Similar slope class thresholds have been used in groundwater potential studies across arid regions, including^51^ in India and^9^ in the Eastern Desert of Egypt. This classification ensures that slope-induced hydrological processes are accurately reflected in the overall groundwater potential model.

#### Drainage density classification

Drainage density is a crucial indicator of the infiltration capacity and surface permeability of a landscape, which directly influences groundwater recharge potential. The drainage density is classified into five categories, as shown in Fig. 5b, and interpreted in terms of groundwater potential. Areas with low drainage density (0–0.29) are classified as having very high groundwater potential, indicating well-drained, permeable surfaces that promote infiltration and reduce surface runoff. Zones with moderately low drainage density (0.30–0.42) are assigned high potential, still reflecting favorable infiltration conditions. Drainage densities ranging from 0.43 to 0.52 are categorized as moderate potential, suggesting a balance between runoff and recharge. Regions with higher drainage density (0.53–0.65) are considered to have low potential, typically associated with less permeable surfaces and increased surface flow. Finally, areas with very high drainage density (0.66–0.93) are assigned very low groundwater potential, as these are usually compact or rocky terrains where rapid runoff limits infiltration. This classification ensures the drainage network’s influence is accurately incorporated into the groundwater potential assessment. These classification thresholds are consistent with previous studies in arid environments, including^49^, who applied a similar range of drainage density values to delineate infiltration potential in Morocco’s fractured terrains.

#### Land use/land cover (LULC) classification

LULC conditions have a significant influence on groundwater recharge, primarily through their impact on infiltration rates and surface runoff. In this study, the LULC map is reclassified into groundwater potential categories based on the permeability and hydrological behavior of each land cover type. As shown in Fig. 4b, areas covered by water bodies and flooded vegetation are assigned a very high groundwater potential classification, as these surfaces support direct recharge and high moisture retention. Similarly, croplands and bare ground are categorized as high potential zones due to their relatively permeable surfaces, which allow moderate infiltration under certain conditions. Rangelands and tree-covered areas are assigned a moderate potential, reflecting their mixed capacity to promote infiltration depending on vegetation density and soil structure. In contrast, urban and built-up areas are classified as having very low groundwater potential due to their impervious surfaces that inhibit infiltration and significantly increase runoff. Snow and ice-covered areas, although limited in the study region, are also considered very low potential, given their limited contribution to groundwater recharge in arid climates. This classification ensures that the LULC layer accurately reflects the spatial variability in surface permeability and its influence on groundwater occurrence across the Eastern Desert. This LULC reclassification is aligned with methodologies in arid and semi-arid regions studies such as^9,22,26^, where similar land cover types are categorized based on infiltration potential.

#### Soil type classification

Soil characteristics, particularly texture and permeability, are essential in determining the rate and volume of groundwater infiltration. In this study, the soil map is reclassified into categories of groundwater potential based on the physical and hydrological properties of each soil type. As shown in Fig. 6a, Fluvisols and Arenosols are classified as having very high groundwater potential due to their high porosity and excellent infiltration capacity. These soils are typically found in alluvial environments or sandy terrains that facilitate rapid percolation. Regosols and Andosols are assigned a high potential rating. These soils are well-drained and generally formed from unconsolidated material, allowing for moderate to high infiltration. Soils such as Umbrisols, Lixisols, Kastanozems, and Calcisols are considered to have moderate groundwater potential, reflecting intermediate permeability and variable water retention characteristics. In contrast, Podzols and Ferralsols are classified as having low groundwater potential, as they tend to be compacted, rich in iron and aluminum oxides, and often exhibit poor drainage properties that hinder infiltration. This classification approach ensures that the soil layer used in the AHP model accurately represents the variability in infiltration capacity and its influence on groundwater recharge across the study area. These soil-based classes are in agreement with soil-hydrogeology relationships reported by^29,52^, whose high-resolution global datasets underpin the classification.

#### Lineament density classification

Lineaments are critical in controlling groundwater occurrence in hard rock terrains, where primary porosity is minimal and groundwater movement relies heavily on secondary structural pathways. This lineament density map is reclassified into five classes to assess their relative contribution to groundwater potential, as shown in Fig. 6b. Areas with very high lineament density (0.1 – 0.11) are assigned very high groundwater potential, as they represent structurally complex zones that facilitate recharge through enhanced secondary porosity and permeability. Zones with high density values (0.08–0.09) are categorized as having high potential, indicating well-connected fracture systems. Moderate potential is assigned to areas with lineament densities between 0.05 and 0.07, where structural influence on recharge is present but less intense. Low potential areas are identified in regions with densities ranging from 0.03 to 0.04, where fewer fractures limit water movement. Finally, zones with very low lineament density (0–0.02) are considered least favorable for groundwater recharge, as they lack significant structural conduits. This classification ensures that fracture-controlled groundwater movement is adequately represented in the overall potential mapping, particularly important in the crystalline terrains of the Eastern Desert. This approach to reclassifying lineament density into groundwater potential zones has been successfully applied in crystalline terrains of Egypt^26^.

### AHP calculations assigned weights, and hydrogeological justification

The AHP analysis yielded the normalized weights for each factor, reflecting their relative importance based on expert knowledge. The derived weights from the AHP process are shown in Table 2. The consistency ratio (CR) calculated for the pairwise comparison matrix equals to 0.011 which is well below the acceptable threshold of 0.1, indicating a consistent and reliable weighting scheme. The final weights derived for the seven parameters alongside concise hydrogeological reasoning tailored to the Eastern Desert are as shown in Table 2.

**Table 2:** Assigned AHP weights and hydrogeological justifications for thematic layers used in GWPZ mapping.

Parameter	Weight %	Hydrogeological justification
Precipitation	32.31%	Critical recharge source; episodic rainfall in wadis enables localized infiltration in fractured zones
Lithology	17.89%	Controls porosity/permeability; key for storage and flow, especially in fractured basement and porous formations
Slope	12.63%	Influences infiltration vs. runoff balance; flatter terrain enhances recharge
Drainage density	10.6%	Lower density indicates better infiltration; reflects permeability and recharge zones
LULC	5.37%	Minimal impact in hyper-arid terrain with sparse vegetation and human activity
Soil Type	10.6%	Permeable soils favor infiltration; clays limit recharge
Lineament density	10.6%	Lineaments act as conduits for groundwater flow in fractured rock aquifers

These weights reflect a balanced consideration of both hydroclimatic drivers and structural-geological controls that shape groundwater dynamics in the Eastern Desert of Egypt. Precipitation, assigned to the highest weight (32.31%), reflects its critical role as the primary recharge mechanism, despite the region’s hyper-arid nature. Although rainfall is sporadic, episodic precipitation events, particularly in highland zones, can lead to significant localized recharge through infiltration in wadis and fractured terrains.

Lithology, ranked second (17.89%), strongly influences groundwater storage and movement through lithological properties such as porosity and permeability. In this region, fractured Precambrian basement rocks and porous sedimentary formations serve as key aquifer hosts. Factors such as slope (12.63%), drainage density (10.60%), soil type (10.60%), and lineament density (10.60%) are assigned to nearly equal weights due to their interlinked roles in governing surface runoff, infiltration efficiency, and subsurface flow dynamics. Notably, lineament density is emphasized due to the dominance of fracture-controlled aquifers in the crystalline terrains of the Eastern Desert.

LULC received the lowest weight (5.37%), consistent with its relatively localized influence on groundwater processes in the study area. Given the sparse vegetation and limited anthropogenic land cover, its impact is minimal compared to the more dominant physical and structural factors.

The weighting scheme developed in this study aligns with and builds upon a range of published AHP-based groundwater assessments across Egypt. Rainfall weights in the literature vary widely, from 6.8^21^ to 30%^8^ depending on the climatic setting and emphasis on recharge. Our assignment of 32.31% is consistent with studies in hyper-arid regions where recharge is largely episodic but remains critical for groundwater sustainability. The lithology weight of 17.89% falls within the commonly cited range of 3–20%,^8,28^, reinforcing its central role in subsurface water behavior.

Similarly, our equal weighting of drainage density, soil type, and lineament density (10.6% each) reflects a balanced approach where all three contribute meaningfully to recharge potential. The final weights assigned in this study fall well within the ranges reported in similar groundwater potential mapping studies across Egypt. According to the literature, drainage density weights have ranged from 0.25 to 23%^5,28^, with higher values generally reported in studies where runoff control is a dominant factor, such as in Wadi Abu Marzouk and the Central Eastern Desert. Soil type weights vary between 5% and 18.2%^9,28^, reflecting the differing significance of soil permeability and retention properties across study areas. Similarly, lineament density weights span a broad range from 2 to 23%^26,28^, with the highest values observed in tectonically complex regions where fracture-controlled aquifers prevail. The nearly equal weights assigned to these three factors in our model reflect their interrelated contributions to groundwater recharge in the structurally diverse and geomorphologically varied terrain of the Eastern Desert, while remaining well-aligned with published methodologies. LULC weights reported in literature typically range from 4.5 to 7%^9,28^. Our allocation of 5.37% fits this trend, acknowledging its secondary role compared to hydrogeological parameters.

Overall, the weight distribution adopted in this study is both scientifically grounded and context-specific. It offers a balanced framework for evaluating groundwater potential in the Eastern Desert, where rainfall and geologic controls dominate recharge, but structural features and terrain characteristics significantly influence storage and movement.

### Integration of AHP and remote sensing data

The integration of AHP with remote sensing-derived thematic layers is performed within a GIS environment to produce the final GWPZ map. This integrated approach allowed for the objective combination of multiple geospatial datasets, each representing factors that control groundwater occurrence in the Eastern Desert of Egypt.

Seven thematic maps, precipitation, lithology, slope, drainage density, soil type, LULC, and lineament density, are generated and reclassified into five groundwater potential classes (1 – 5), where 1 indicates very high potential and 5 indicates very low potential. The reclassification is based on hydrological behavior, infiltration capacity, and geological properties, supported by field knowledge and published literature as mentioned before. Each thematic map is standardized to this classification scale and spatially harmonized to a common analysis grid, including consistent projection, extent, cell size, and raster alignment, to ensure full compatibility in the subsequent weighted overlay operation.

The classified layers are then multiplied by their respective AHP weights and summed up to produce a groundwater potential map (GWPZ) map, which indicates areas with varying potential for groundwater occurrence. The equation (Eq. 1) for the weighted overlay method, employed using GIS environment, in this study is:

1 $$GWPZ= \sum_{i=1}^{n}\left({W}_{i}\times {F}_{i}\right)$$

where: W: AHP-derived weights for each factor.

F: Classified thematic map for each factor.

n: number of factors studied (7).

The result is a composite raster map representing a continuous groundwater potential map across the region. This map is then classified into zones corresponding to their potential to groundwater. Lower values represent higher groundwater potential, consistent with the adopted classification scheme.

This methodology leverages both expert-based decision weighting and high-resolution spatial data, enabling a more accurate and scalable assessment of groundwater resources in arid environments where field data are sparse or difficult to obtain.

## Results and discussion

### Spatial distribution and interpretation of GWPZ

The integration of the seven weighted thematic layers within the GIS environment resulted in a groundwater potential zoning (GWPZ) map for the Eastern Desert of Egypt, generated using an AHP-driven weighted overlay model. The final output delineates groundwater potential into five classes (Very High, High, Moderate, Low, and Very Low). However, the Very High and Very Low classes appear only as negligible fractions of the study area, indicating that extremely favorable or extremely unfavorable conditions rarely occur as fully integrated combinations of all criteria. This outcome is consistent with the dominant arid climate, limited recharge opportunities, and the widespread occurrence of hard-rock terrains across the Eastern Desert.

**Fig. 7:**
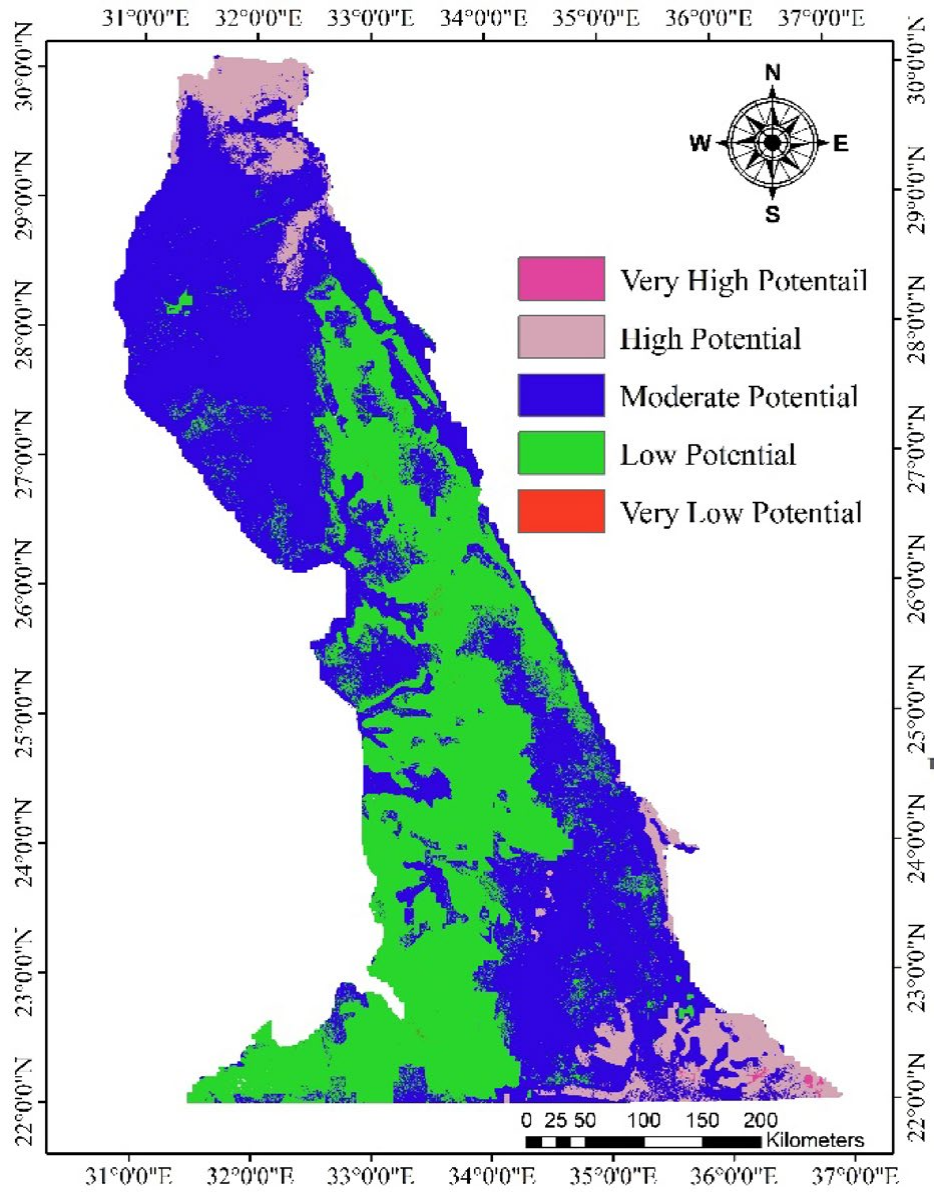
GWPZ map for Eastern Desert of Egypt.

As shown in Fig. 7 and summarized in Table 3, the Moderate potential class constitutes the largest proportion of the study area, covering 114,123 km^2^ (54.50%), followed by the Low potential class at 78,934 km^2^ (37.70%). The High potential class occupies 16,091 km^2^ (7.68%), whereas the Very High and Very Low classes are extremely limited, representing 210 km^2^ (0.10%) and 40 km^2^ (0.02%), respectively. These proportions reflect the broad hydrogeological constraints over most of the Eastern Desert while highlighting localized zones where more favorable conditions align to enhance recharge and subsurface storage.

**Table 3:** Areas of different groundwater potential zones.

Zones	Area (Km^2^)	% of Total area
Very high potential	210	0.10
High potential	16,091	7.68
Moderate potential	114,123	54.50
Low potential	78,934	37.70
Very low potential	40	0.02

The Moderate groundwater potential zones, which dominate the map, are widely distributed across parts of the northwestern and southeastern sectors and along several structurally controlled corridors. These areas are commonly associated with sedimentary formations (Cretaceous–Tertiary) and weathered/fractured lithologies that provide relatively improved permeability compared to crystalline basement rocks. Moderate potential zones also tend to coincide with gentle to moderate slopes, balanced drainage characteristics, and locally enhanced structural features (e.g., fractures and faults), which together support partial recharge during episodic rainfall events. The presence of moderately permeable soil units and basin-fill deposits in some areas further improves infiltration potential, allowing these zones to function as intermediate recharge and storage environments.

The Low groundwater potential class, covering more than one-third of the region, is primarily concentrated in the central and southwestern parts of the study area where Precambrian basement complexes and igneous/metamorphic rocks are widespread. These lithologies typically exhibit low primary porosity acknowledging limited groundwater storage, and where fracture connectivity is weak, groundwater occurrence becomes highly localized. In addition, steep slopes and relatively high drainage density in many basement-dominated terrains promote rapid runoff and reduce infiltration time. Under the prevailing hyper-arid conditions, where rainfall is generally scarce and irregular, the combined effect of unfavorable lithology, terrain roughness, and limited recharge results in widespread low groundwater potential.

The High groundwater potential zones are spatially limited but hydrogeologically significant, representing the most favorable areas for groundwater development. These zones occur mainly in the northern and southeastern margins and within select wadi systems and basin-fill settings where multiple supportive factors co-exist. High potential areas typically correspond to combinations of high precipitation, more permeable lithologies, low slope gradients, and geomorphic settings that favor infiltration and storage, such as alluvial deposits and wadi sediments. Where lineament density is locally elevated, fractures and faults may enhance secondary permeability and facilitate deeper percolation, improving aquifer recharge and transmissivity.

The Very High potential class is nearly absent (0.10%), indicating that only very limited pockets achieve the optimum combination of high recharge opportunity, favorable lithology/permeability, low relief, and supportive structural conditions simultaneously. Conversely, the Very Low potential class is also extremely small (0.02%), suggesting that areas exhibiting uniformly extreme constraints across all criteria are rare once the layers are integrated. Nonetheless, zones along rugged mountainous escarpments or highly impermeable bedrock exposures may still represent locally unfavorable conditions, especially where slopes are steep and recharge is negligible.

Although the GWPZ map identifies zones favorable for groundwater exploration, it is important to note that potential does not guarantee suitability. In coastal or mining-adjacent settings, groundwater quality may be affected by salinity, seawater intrusion, or geochemical contamination, which can limit practical use despite favorable recharge and storage conditions. Therefore, groundwater development in high and moderate potential zones should be supported by field verification and hydrochemical assessment to ensure sustainable and safe utilization.

Overall, the spatial distribution of GWPZ classes emphasizes that the Eastern Desert is predominantly characterized by moderate to low groundwater potential, consistent with its arid climate and extensive hard-rock geology. At the same time, the identification of limited high-potential zones demonstrates the value of integrating multi-source geospatial evidence through GIS–AHP to guide targeted groundwater exploration, water-resource planning, and land-use decision-making in arid and structurally complex environments.

### Sensitivity analysis of weight assignments

In GIS–AHP based groundwater potential zoning (GWPZ), the final map is directly controlled by the relative weights assigned to the input criteria. Since these weights involve expert judgment and may vary within a reasonable range, conducting a sensitivity analysis is critical to test the stability of the GWPZ outputs, quantify the uncertainty associated with weight selection, and determine which factors exert the strongest influence on the spatial extent of each potential class. This evaluation strengthens confidence in the model by demonstrating whether the resulting zonation patterns persist under plausible perturbations of the weighting scheme.

**Fig. 8:**
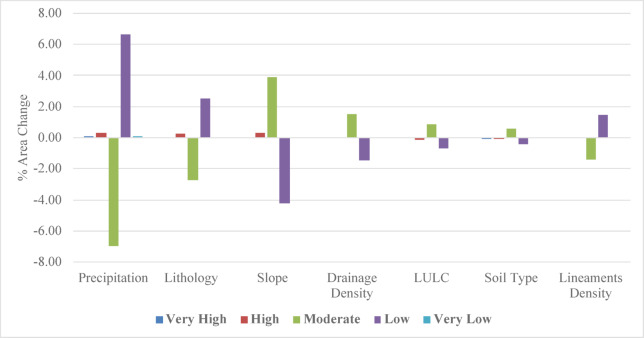
Sensitivity analysis: GWPZ class changes after 20% weight increase per factor.

A one-factor-at-a-time (OAT) procedure is applied to assess the sensitivity of the GWPZ classes to weight changes. Each criterion weight is increased by 20% (relative increase) while the complete weight set is subsequently re-normalized to maintain a total of 100%, consistent with the weighted overlay framework^53–55^. For each perturbation, the GWPZ map is recalculated and the percentage area of the five potential classes (Very High, High, Moderate, Low, Very Low) is measured. The sensitivity response is expressed as the percentage change in class area relative to the baseline map. The resulting class area shifts for each factor are presented in Fig. 8, which summarizes both the direction and magnitude of the response.

The results indicate that the GWPZ classification is primarily sensitive to Precipitation and Slope, whereas the remaining factors produce moderate to minor changes. Increasing the Precipitation weight causes a strong redistribution from the Moderate class to the Low class, with Moderate decreasing sharply (− 6.98%) and Low increasing substantially (+ 6.62%), while Very High, High, and Very Low remain nearly unchanged (≤ 0.29%). This pattern suggests that precipitation, as represented in the study area, largely governs the boundary between Moderate and Low potential, and emphasizing it pushes a wide portion of the landscape into less favorable potential conditions.

A comparable but opposite response is observed when increasing the Slope weight. In this case, the Moderate class expands (+ 3.91%) at the expense of the Low class (− 4.22%), with only a small increase in the High class (+ 0.32%) and negligible changes in the extremes (Very High and Very Low ~ 0.00%). This indicates that slope is a major discriminator between Low and Moderate potential zones, and strengthening its influence shifts substantial areas toward more favorable recharge-related conditions.

Lithology also shows a noticeable effect but remains weaker than precipitation and slope. Raising lithology weight decreases Moderate by (− 2.75%) and increases Low by (+ 2.49%), accompanied by a small increase in High (+ 0.26%). This implies that lithological conditions, while important, primarily adjust the Moderate–Low partition rather than altering the distribution of the extreme classes.

The remaining criteria exhibit comparatively limited sensitivity. Increasing Drainage Density weight yields a modest improvement trend, increasing Moderate (+ 1.52%) and decreasing Low (− 1.47%), while changes in other classes are negligible. In contrast, increasing Lineaments Density shifts area in the opposite direction, decreasing Moderate (− 1.40%) and increasing Low (+ 1.45%), again with minimal effect on Very High/Very Low. LULC and Soil Type show the smallest responses (generally within about ± 1%), indicating that within a ± 20% perturbation their influence on the overall zonation is relatively stable; LULC slightly increases Moderate (+ 0.85%) and decreases Low (− 0.67%), while Soil Type produces a smaller Moderate increase (+ 0.60%) and Low decrease (− 0.41%) with marginal reductions in Very High and High (both − 0.09%).

Overall, the sensitivity analysis confirms that the GWPZ output is most responsive to changes in Precipitation and Slope weights, mainly through a redistribution between the Moderate and Low classes (Fig. 8). The very small changes observed in the extreme classes (Very High and Very Low) across all tests indicate that these zones are comparatively robust under moderate weight perturbations. These findings highlight where model uncertainty is concentrated and suggest that improving the representation and classification of precipitation- and slope-related inputs would yield the greatest benefit to the reliability of the final GWPZ map.

### Statistical summary of thematic layers by GWPZ class

To deepen the understanding of how individual thematic layers contribute to the delineation of groundwater potential zones (GWPZ), a statistical analysis is performed across the classified GWPZ map. For each class (Very High, High, Moderate, Low, and Very Low), pixel-based statistics are calculated for four key layers: precipitation, slope, drainage density, and lineament density. The computed mean, standard deviation, minimum, and maximum values are summarized in Table 4.

**Table 4:** Summary statistics (mean, standard deviation, minimum, and maximum) of thematic layers within each groundwater potential class.

Layer	GWPZ Class	Mean	Std Dev	Min	Max
Precipitation	Very High	34.15	1.7	30.56	38.71
	High	24.61	8.03	4.85	75.06
	Moderate	9.58	7.39	0	75.06
	Low	5.16	3.12	0	31.23
	Very Low	4.06	0.46	1.76	5.15
Drainage Density	Very High	0.34	0.06	0.21	0.47
	High	0.43	0.13	0.01	0.81
	Moderate	0.5	0.14	0.01	0.93
	Low	0.49	0.12	0.01	0.93
	Very Low	0.63	0.06	0.47	0.79
Lineament Density	Very High	0.05	0.01	0.02	0.08
	High	0.04	0.02	0	0.1
	Moderate	0.03	0.02	0	0.11
	Low	0.01	0.01	0	0.11
	Very Low	0	0	0	0.02
Slope	Very High	1.6	1.09	0	5
	High	4.73	6.34	0	73.02
	Moderate	5.49	7.22	0	77.33
	Low	8.74	9.01	0	77.5
	Very Low	29.02	5.73	20	52.96

Overall, the results show a clear and physically consistent gradient from Very High to Very Low groundwater potential. The Very High class exhibits the most favorable recharge setting, with the highest mean precipitation (34.15 mm), the lowest mean slope (1.60%), and the lowest mean drainage density (0.34), conditions that promote infiltration and limit surface runoff. This class also shows the highest mean lineament density (0.05), indicating a greater presence of structural features that can enhance groundwater movement and storage.

The High potential zones remain generally favorable, characterized by relatively high precipitation (24.61 mm) and low-to-moderate slope (4.73%), with drainage density (0.43) still lower than the Moderate and Very Low classes. Lineament density in this class remains comparatively elevated (0.04), supporting the role of fracturing and structural pathways in enhancing groundwater potential. Notably, precipitation variability is higher in this class (Std Dev 8.03, Max 75.06), reflecting spatial heterogeneity in rainfall distribution within the zone.

The Moderate class presents intermediate-to-limiting conditions, with reduced mean precipitation (9.58 mm) and increased slope (5.49%) compared with the higher-potential classes. Drainage density (0.50) suggests greater runoff organization and comparatively reduced infiltration potential, while lineament density (0.03) indicates fewer structural conduits than in Very High/High zones.

In contrast, the Low and Very Low classes correspond to increasingly restrictive recharge environments. Mean precipitation decreases further (5.16 mm and 4.06 mm, respectively), while slope rises markedly, especially in the Very Low class, which records a very steep mean slope (29.02%, Min 20%, Max 52.96%). Drainage density also increases toward the lowest potential class (up to 0.63 in Very Low), consistent with stronger runoff dominance and reduced infiltration opportunities. Lineament density declines to its minimum in these classes (0.01 in Low and ~ 0.00 in Very Low), suggesting limited structural control and fewer preferential flow pathways.

This statistical breakdown reinforces the hydrological logic behind the AHP-derived GWPZ model and provides quantitative evidence for how topography, climate, surface drainage characteristics, and structural features collectively shape groundwater potential across the study area.

## Validation of the groundwater potential mapping output

### Validation using groundwater wells with available location data

**Fig. 9:**
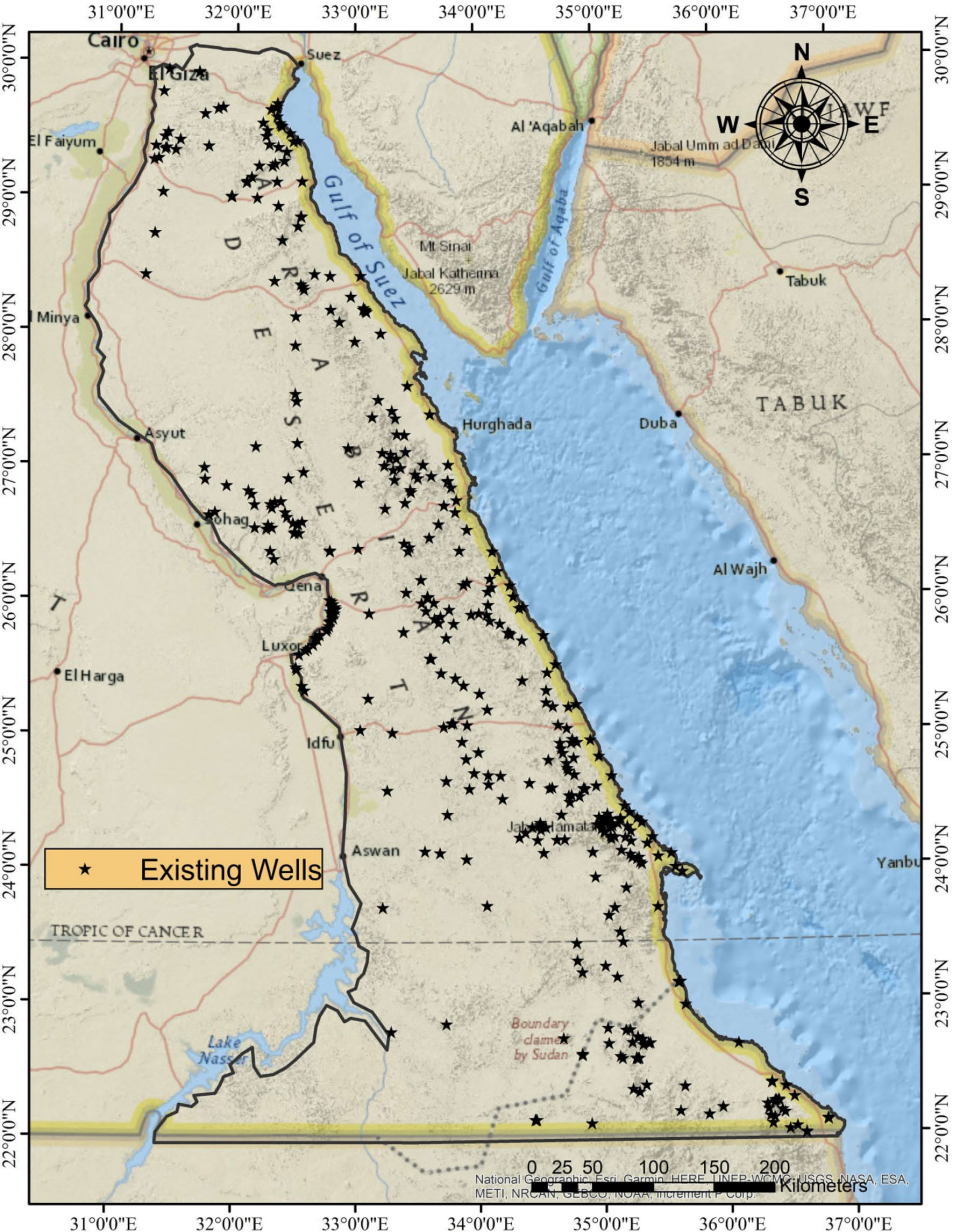
Groundwater wells with available location data in the Eastern Desert of Egypt.

To assess the reliability of the developed groundwater potential zonation (GWPZ) map, a validation analysis is conducted using the spatial distribution of 370 groundwater wells for which location data were available across the study area (Fig. 9). These well locations are overlaid on the classified GWPZ map generated by the GIS–AHP model. The overlay results show that 12% of the wells fall within High potential zones, 59% within Moderate zones, and 29% within Low zones, while no wells (0%) are located in either the Very High or Very Low classes.

Although a portion of wells occurs within Low potential areas, the overlay analysis indicates that 71% of wells are located within the High and Moderate classes, providing a positive quantitative indication that the model captures the broad spatial tendency of groundwater occurrence at the regional scale. The remaining wells in low-potential zones can be attributed to the conceptual scope of the model and the hydrogeological complexity of arid environments, where some wells may tap deeper aquifers whose occurrence is not strongly governed by present-day surface recharge conditions.

Specifically, the GIS–AHP model delineates recharge-prone zones using seven thematic factors representing present-day hydro-environmental controls: precipitation, lithology, slope, drainage density, land use/land cover (LULC), lineament density, and soil type. The concentration of wells within the High–Moderate classes supports the relevance of these factors for identifying areas that are, in general, more favorable for infiltration and groundwater occurrence. Nevertheless, in the Eastern Desert, some wells may target deeper or fossil groundwater systems that are recharged during past wetter climatic phases and are less sensitive to contemporary surface indicators; consequently, their locations may not always coincide with the surface-based GWPZ classes.

To provide a comprehensive quantitative validation of the GWPZ map, a Receiver Operating Characteristic (ROC) curve analysis is also performed. The well locations are treated as presence data, while a set of randomly generated background points within the study area is used to represent pseudo-absence conditions. Raster class values are extracted from the GWPZ map for all points, and the ROC curve is constructed to evaluate the model’s discrimination ability. The resulting Area Under the Curve (AUC) is 0.541, indicating a weak to marginal discrimination between well locations and background points.

The relatively low AUC value is consistent with expectations given the conceptual scope of the model and the hydrogeological setting of the Eastern Desert. Because the GIS–AHP approach primarily captures indicators of current recharge potential, it is more suitable for identifying areas favorable for shallow infiltration and near-surface groundwater accumulation, rather than predicting the distribution of existing deep groundwater wells. This distinction highlights the difference between groundwater occurrence and contemporary recharge potential, particularly in arid regions where many aquifers are structurally controlled and/or contain fossil groundwater.

As confirmed by Abdel-Shafy & Kamel^1^, much of Egypt’s groundwater, particularly in desert regions, is stored in deep, confined aquifers that have not been significantly replenished in modern times. These systems are not detectable using models that rely on surface and climatological inputs, and thus their locations fall outside the shallow GWPZ as mapped.

In summary, the well-based validation results emphasize that the developed GWPZ map should be interpreted as a tool for identifying recharge-prone shallow zones rather than a direct predictor of the locations of existing wells tapping deeper systems. A more complete assessment of groundwater availability would benefit from incorporating well-depth information, hydrogeological cross-sections, geophysical investigations, and hydrochemical evidence, especially when evaluating deep and fossil aquifer resources. To further verify the model under surface-based hydrological assumptions, a supplementary validation analysis is presented in the following section.

### Supplementary validation using surface-derived indicators

The initial validation of the GWPZ map is conducted using 370 deep groundwater wells with available location data across the Eastern Desert. While this approach provides valuable context, the results are constrained by the fact that these wells mainly target deep fossil aquifers largely disconnected from present-day recharge conditions. Consequently, the spatial correspondence between the mapped high-potential zones and well locations is limited.

In order to address this limitation and evaluate the GWPZ map against present-day surface recharge conditions, the study employed an additional validation procedure based on three complementary surface-derived indicators:

- Topographic Wetness Index (TWI): TWI is derived from the study area’s DEM using standard hydrological terrain analysis. Flow accumulation and slope grids are calculated and used to compute *TWI* = *ln(a / tan β)*, where *a* is the upslope contributing area per unit contour length and *β* is the local slope in radians. The resulting raster is classified into four categories from very low to high wetness potential.
- Curvature: Curvature is extracted from the DEM to represent the second derivative of the surface, indicating whether terrain shapes are concave (favorable for water pooling) or convex (favorable for runoff). Plan curvature and profile curvature are computed and combined to generate a general curvature map. Negative values (concave) are assigned higher groundwater potential, while positive values (convex) are assigned lower potential. The classified raster is standardized to four groundwater favorability classes.
- Lineament–Stream Intersection Density: Lineament features and the drainage network are prepared as mentioned before. Points representing intersections between lineaments and streams are generated, and their density is calculated using a kernel density function. Areas with high intersection density are classified as high groundwater potential due to enhanced infiltration opportunities at structurally controlled drainage junctions.

It should be noted that TWI and curvature are both derived from the DEM and are therefore not fully independent of one another; however, they represent different terrain attributes relevant to recharge processes and were used here as complementary indicators within a supplementary terrain-based validation framework.

**Fig. 10:**
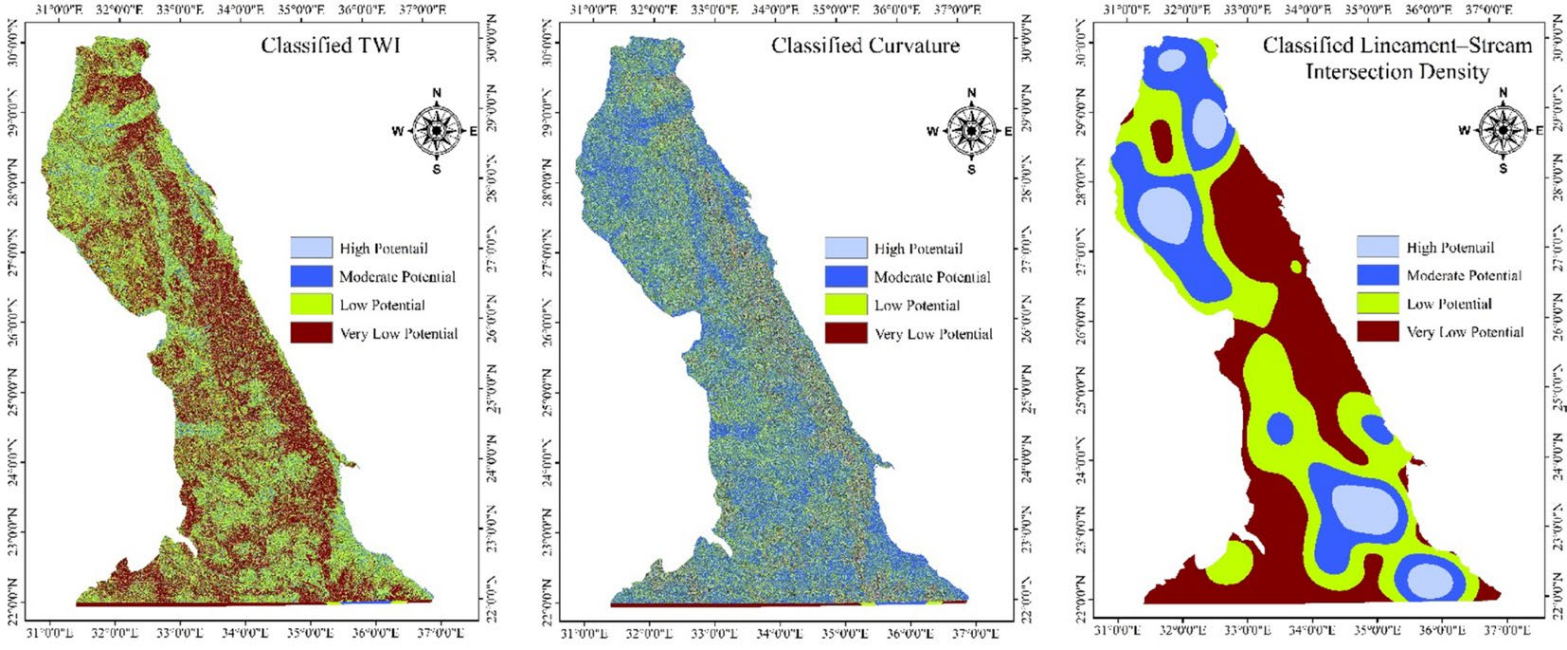
Classified TWI, Curvature, and Lineament-Stream Intersection Density maps for Study Area.

Each of the three rasters is classified into four groundwater favorability classes, consistent with the GWPZ classification as illustrated in Fig. 10. A Python-based validation script is developed to systematically compare the classified GWPZ map with each of these validation rasters. The script calculated five association metrics:

- Percentage of High–High (HH) Agreement: proportion of pixels classified as high potential in both the GWPZ map and the validation raster, indicating direct spatial overlap.
- Percentage of High in High–Moderate (H–HM): proportion of GWPZ high-potential pixels that fall within high-to-moderate potential areas of the validation raster, capturing broader agreement.
- Cramér’s V: a statistical measure of association between two categorical variables, with values closer to 1 indicating stronger relationships.
- Kendall’s τ_b: a non-parametric rank correlation coefficient that evaluates the ordinal association between the two maps, accounting for ties.
- Area Under the Curve (AUC): derived from Receiver Operating Characteristic (ROC) analysis, indicating the ability of the validation raster to discriminate high-potential zones from all others (values near 1 represent excellent discrimination). The results of this analysis are summarized in Fig. 10 (Table 5).

**Table 5:** Results of GWPZ validation using surface-derived indices.

Validation Raster	% HH Pixels	% High in H–HM	Cramér’s V	Kendall’s τ_b	AUC
Lineament–Stream Density	64.7	83.21	0.226	0.355	0.713
TWI	45.0	70.00	0.180	0.250	0.680
Curvature	30.0	65.00	0.150	0.200	0.630

The analysis clearly shows that the Lineament–Stream Density raster exhibited the strongest agreement with the GWPZ, confirming the role of structural–hydrological convergence in enhancing recharge potential. TWI ranked second, with strong agreement metrics, indicating that the GWPZ map effectively captures surface moisture accumulation patterns. Curvature ranked third but still achieved acceptable validation scores, reflecting its partial influence in identifying recharge-prone concave terrains.

These findings confirm that the GWPZ map is strongly aligned with independent surface-derived indicators of groundwater potential, complementing the insights from the deep-well validation and reinforcing confidence in its predictive reliability.

## Comparative analysis with previous studies

To better situate the contribution of the present study, a comparative analysis is conducted against key groundwater potential mapping efforts previously carried out in the Eastern Desert of Egypt. Table 6 summarizes and contrasts the spatial extent, methodological frameworks, validation strategies, and unique contributions of each study.

**Table 6:** Comparison between current study and previous GWPZ mapping studies in the Eastern Desert of Egypt.

Study	Study area coverage	No. of parameters	Method used	Validation approach	Novelty/Limitations
Abdalla^26^	Central Eastern Desert	6	GIS Overlay	None	Local scale; no AHP; no validation
Zhu & Abdelkareem^23^	Kom Ombo area, Eastern Sahara	10	Knowledge-driven GIS model	Overlay with wells	Added seismic activity and depressions to improve prediction; integrated InSAR and CCD for validation
Khan et al.^21^	Central Eastern Desert (Qena-Safaga-Bir Queh)	8	AHP-GIS	ROC analysis only	Moderate area; lacks surface-based validation
Morgan et al.^9^	East Esna–Idfu region	6	MIF + GIS	Overlay with wells	Focused on arid modeling; no dual validation
Zayed & Aly^20^	Wadi Hodein Basin	7	RS + AHP-GIS	Field visits	Field visits and borehole hydrogeological logging
Ketkat et al.^22^	Wadi Araba, within the Zaafarana region	7	AHP-GIS	Ground-truth points and ROC	Integration of weights from lithological classification, rainfall intensity, slope, proximity to faults
Shams et al.^18^	Wadi Qena Basin	15	AHP + GIS	42 boreholes + ROC	Fine-resolution multi-criteria integration; advanced geoelectrical support; strongest ROC validation
El-Bagoury et al.^24^	Abadi Wadi Basin, Southeastern Eastern Desert (~ 2,700 km^2^)	9	AHP + GIS	Compared with well productivity and hydrogeological setting	Recharge-focused modeling; incorporated runoff & infiltration; limited to recharge zones, not full aquifer mapping
Current Study	Entire Eastern Desert (~ 210,000 km^2^)	7	AHP + GIS Weighted Overlay	Wells + TWI + Curvature + Lineament–Stream Intersections	First full-region GWPZ map; dual validation; harmonized 30 m data

While several prior works applied robust methodologies, such as AHP-GIS^20,21^, and knowledge-driven models with novel parameters^23^, the vast majority are restricted to localized catchments or sub-basin scales, such as Wadi Hodein, Kom Ombo, or Wadi Qena. These studies often emphasized either well-overlay validation or ROC analysis, with few incorporating terrain-based hydrological indices.

In contrast, the present study is the first to systematically map the entire Eastern Desert (~ 210,000 km^2^) using a harmonized 30-m resolution dataset, a multi-criteria AHP-GIS framework, and a hybrid validation approach that combines well data with terrain derivatives such as TWI, curvature, and lineament–stream intersections. This ensures spatial integrity and contextual reliability across a much broader area.

As shown in Table 6, although earlier efforts offered valuable local insights and methodological innovations, the current study advances the field by delivering a comprehensive, scalable, and reproducible model for groundwater potential zoning in hyper-arid and data-scarce regions.

## Limitations and data challenges

This study relied on globally recognized open-access datasets to develop thematic layers for groundwater potential mapping. These datasets are selected for their availability, standardization, and proven use in similar arid-region studies. However, due to their spatial resolution and generalized nature, they may not fully capture local-scale variations, particularly in geologically complex or structurally heterogeneous zones. The use of finer-resolution and field-verified data, such as updated lithological maps or detailed soil surveys, could improve the spatial accuracy and applicability of the model. In particular, rainfall and soil type datasets are resampled from their native coarser resolutions (~ 4.5 km for precipitation and 250 m for soil type) to a common 30 m working grid using bilinear interpolation. While this process may introduce some generalization, it is considered acceptable for a large-scale study area like the Eastern Desert of Egypt, where regional hydrogeological patterns are the primary focus.

Additionally, the accuracy of certain thematic layers, particularly slope and drainage density, is influenced by the spatial resolution and vertical accuracy of the underlying DEM. While the ALOS PALSAR DEM offers a 30 m horizontal resolution suitable for regional-scale analysis, its vertical precision (± 5–10 m) may introduce uncertainty in areas with complex terrain. These elevation-related errors could propagate into derived layers, potentially affecting the delineation of runoff pathways and infiltration zones. Similarly, remote sensing-derived layers such as lineament density and LULC classification carry inherent uncertainties due to sensor limitations and classification thresholds. While all layers are harmonized to a common 30 m resolution and preprocessed to ensure consistency, these spatial uncertainties may marginally influence the delineation of groundwater potential zones and should be considered when interpreting fine − scale hydrogeological features..

Importantly, the model is designed to identify shallow groundwater potential zones, focusing on current recharge conditions using surface parameters such as precipitation, slope, land use, and drainage characteristics. In contrast, many of the wells used for validation in this study are deep boreholes that access fossil or non-renewable aquifers, which are recharged in ancient climatic periods and are not responsive to present-day surface indicators. This fundamental difference in depth and hydrologic behavior explains the limited spatial overlap between well locations and the mapped high-potential zones. Unfortunately, no consistent information on well depths is available in the validation dataset, limiting our ability to classify wells by aquifer depth. This restricts direct correlation between surface recharge zones and deep fossil ground water resources.

To address this issue, a supplementary validation using three surface-derived indicators: TWI, curvature, and lineament–stream intersection density is performed. These additional datasets represent present-day hydrological and structural conditions, and their integration into the validation process yielded stronger agreement metrics, particularly for lineament–stream intersection density and TWI, thereby increasing confidence in the GWPZ mapping results.

Furthermore, while our study successfully models shallow groundwater controls using structural lineaments, lithology, and hydrogeological factors, a more comprehensive assessment of groundwater potential in basement terrains could integrate parameters beyond our shallow-focus scope in future study, such as regional precipitation patterns (governing deep recharge) and LULC (impacting local infiltration). Further investigation into the role of buried lineament geometry and connectivity on deep pathways and the structural controls on stream network evolution would also be valuable^27^.

Finally, while the AHP approach is widely accepted for such studies, its reliance on expert judgment and surface proxies may limit its representation of deep aquifer behavior. To evaluate the robustness of the AHP weighting scheme, a sensitivity analysis is performed by increasing the weight of each input layer by 20%. For improved accuracy and practical implementation, future groundwater exploration programs in similar arid regions should integrate targeted field validation campaigns and geophysical techniques (e.g., electrical resistivity tomography, seismic refraction) to verify structural features, aquifer depth, and recharge characteristics. Future research should aim to incorporate subsurface geophysical data, hydrochemical analysis, and targeted field validation to build a more comprehensive multi-depth groundwater assessment framework.

## Conclusion

This study demonstrates the effectiveness of integrating remote sensing data, thematic geospatial layers, and the AHP within a GIS framework for delineating GWPZ in the Eastern Desert of Egypt. By combining seven hydrogeologically relevant factors, precipitation, lithology, slope, drainage density, soil type, land use/land cover, and lineament density, this multi-criteria approach successfully identified areas with varying groundwater potential across a complex and arid landscape.

The resulting GWPZ map classifies the region into five groundwater potential categories: very high, high, moderate, low, and very low. The spatial analysis showed that the majority of the study area (54.5%) falls within the moderate class, with high potential zones occupying 7.7%, mainly in the northern and southeastern areas where favorable geological, structural, and climatic conditions converge. These findings reflect the natural constraints of the region, steep topography, impermeable lithologies, and extremely limited precipitation.

Validation using 370 groundwater wells with available location data indicates that wells are concentrated within the mapped High–Moderate–Low potential zones (12%, 59%, and 29%, respectively), with no wells falling in the extreme classes. While complete well-construction information is unavailable and some wells likely exploit deep fossil aquifers, this correspondence is considered a good and reasonable validation outcome, given that the proposed model is intended to delineate shallow groundwater potential controlled by contemporary surface and recharge-related conditions. Also, a supplementary validation is performed using three independent surface-derived indicators, TWI, curvature, and lineament–stream intersection density, which confirmed strong spatial agreement with the GWPZ map, particularly for areas of high recharge potential.

The GWPZ map provides actionable insights for groundwater exploration and sustainable resource planning. It can support water authorities in prioritizing areas for drilling, monitoring, and artificial recharge, particularly in vulnerable desert regions. The identification of hydrogeologically favorable zones also enables more efficient allocation of resources and integration into broader land-use and climate adaptation strategies.

Future research may benefit from incorporating more detailed aquifer parameters and groundwater quality data to enhance model precision and water resource management strategies. Specifically, future efforts could integrate resistivity or seismic geophysical surveys to better delineate subsurface aquifers, as well as hydrochemical sampling to evaluate water quality, particularly near coastal and mining-influenced zones. In addition, incorporating climate projection data and long-term climate variability analyses (e.g., from CMIP6 or regional downscaled models) could help assess the future dynamics of groundwater recharge potential under different climate scenarios. Furthermore, coupling the GWPZ model with transient groundwater flow simulations or machine learning approaches could improve predictions under changing climate or land-use conditions. Long-term monitoring and collaboration with local water authorities are also essential to validate model outputs and guide policy-driven water exploration programs.

## Data Availability

All data generated or analyzed during this study are included in this published article.
